# Large Impacts of Climatic Warming on Growth of Boreal Forests since 1960

**DOI:** 10.1371/journal.pone.0111340

**Published:** 2014-11-10

**Authors:** Pekka E. Kauppi, Maximilian Posch, Pentti Pirinen

**Affiliations:** 1 Department of Environmental Sciences, University of Helsinki, Helsinki, Finland; 2 Finnish Forest Research Institute (METLA), Vantaa, Finland; 3 Coordination Centre for Effects (CCE), National Institute for Public Health and the Environment (RIVM), Bilthoven, The Netherlands; 4 Climate Service Centre, Finnish Meteorological Institute, Helsinki, Finland; Université Pierre et Marie Curie, France

## Abstract

Boreal forests are sensitive to climatic warming, because low temperatures hold back ecosystem processes, such as the mobilization of nitrogen in soils. A greening of the boreal landscape has been observed using remote sensing, and the seasonal amplitude of CO_2_ in the northern hemisphere has increased, indicating warming effects on ecosystem productivity. However, field observations on responses of ecosystem productivity have been lacking on a large sub-biome scale. Here we report a significant increase in the annual growth of boreal forests in Finland in response to climatic warming, especially since 1990. This finding is obtained by linking meteorological records and forest inventory data on an area between 60° and 70° northern latitude. An additional increase in growth has occurred in response to changes in other drivers, such as forest management, nitrogen deposition and/or CO_2_ concentration. A similar warming impact can be expected in the entire boreal zone, where warming takes place. Given the large size of the boreal biome – more than ten million km^2^– important climate feedbacks are at stake, such as the future carbon balance, transpiration and albedo.

## Introduction

Variations of ambient temperature in boreal forests strongly affect the ecosystem processes [1], [2], [3]. Increased greenness and amplified seasonal changes of CO_2_ have been observed [4], [5], but the improvement of productivity has not been correlated with warming observations. If climatic warming has impacts on forest growth, this may significantly affect the carbon budget, transpiration and forest albedo [6], [7].

Growing degree-days (GDD) are the annual sum of daily temperatures above a pre-defined threshold. GDD combines the effects of favourable temperature on the duration and intensity of growth. Two articles published in the 1980 s projected an increase in the growth of boreal forests under future climate warming [8], [9]. A regression was reported, showing how forest growth is positively correlated with GDD ranging from 700 degree-days and low increment in northernmost boreal forests to 1,350 degree-days and high increment in southern boreal forests [8]. Moreover, the regression was used to explore hypothetical warming scenarios [9]. The eco-physiological mechanisms of this correlation are not fully understood. However, process-based ecosystem models suggest that an accelerating nitrogen cycle is an important, though conceivably not the only mechanism [3].

And indeed, a considerable warming has occurred in northern latitudes since the 1980 s [10]. In Canada, a positive and significant growth response to warming has been reported, based on inventory measurements on 1,267 sites, combining observational data with models, which relate stand growth to stand age [11]. Here we analyse a large amount of data from Finland, collected and compiled by the Finnish Meteorological Institute (FMI) and the Finnish Forest Research Institute (METLA), and apply the empirical methodology used earlier [8]. All boreal forests growing on a land area of 302,000 km^2^ in Finland were covered in the monitoring system. Forest data from Finland are very well documented and have been made available in reference [12]. We describe the study region, derive spatially representative climate trends in terms of Growing Degree Days (GDD), estimate a regression between forest growth and GDD from recent data drawing on the geographic co-variation of the two variables, and finally compare the results with the findings and projections from the 1980 s.

## Materials and Methods

**Figure 1:**
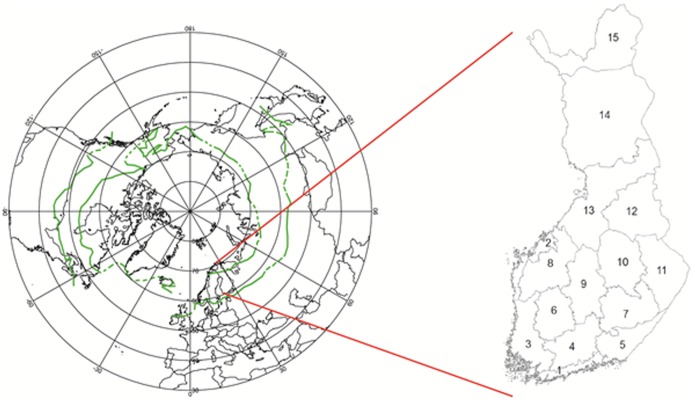
The boreal zone as presented in [9] and the 15 regions of this study located within the boreal biome in Finland.

The method of this research is based on the empirical finding that spatial and temporal variations of forest growth (annual increment) correlate with variations of GDD [8], [9], [13]. The study area is located in Finland, stretching from the southern to the northern limit of the boreal biome and is divided in 15 regions (Fig. 1). The Ahvenanmaa region (Åland Islands) is excluded as it has a temperate character and the forested area is small (about 1,000 km^2^). Region 1 surrounding Helsinki is at the southern edge of the boreal zone, where agriculture and settlements are widespread but, nevertheless, 54% of the land is covered by forests. Region 15 between 68° and 70°N is partly located beyond the Arctic timber line and is the only one where forest land does not predominate as the main land cover type. The distance between the most northern and southern regions is about 900 km.

During 1961–2013 the FMI has recorded daily mean temperature at 120–210 stations across Finland. Station data on the daily mean temperature and the beginning and end of the growing season have been interpolated onto a 10 km ×10 km grid using a kriging method [14], [15]. GDD for each grid point and each year has been calculated using a threshold temperature of +5°C. From these estimates we computed the spatial average GDD for each region and each year. Linear regressions were fitted to these time series.

The 11^th^ National Forest Inventory (NFI) of Finland was carried out in 2009–2012. The sample plots of this inventory provide a rigorous network of measurements, which was distributed over all regions. Forest growth (or annual increment) refers to the accumulation of woody tissue on tree stems during one growing season. A national (and Nordic) convention of the concept ‘forest growth’ was applied, which does not take into account the contribution of the trees lost in natural mortality. However, the growth of recently harvested trees is included into the growth estimate. The concept is similar to ‘gross growth’ as referred to in [11].

The methods of measuring forest increment are well tested, because there has been a persistent economic incentive to obtain accurate information on this natural resource [16]. The number of sample plots measured in the 11^th^ NFI varied between regions from 567 to 4,377 (Table 1). While the total number of plots was large (39,276), an even more important characteristic of the forest inventory system is that the plots were located using a well-tested statistical algorithm [16]. Therefore, the sample represents all forests in each region. Forest growth is directly measured from sample trees observing the width of the five most recent complete tree rings and the stem elongation of the leader shoot during a five-year period preceding the observation. As the sample trees for growth measurements are statistically chosen on each plot, the results represent the growth of all trees growing on forest land in each region. A linear regression was fitted to the data for describing the dependence of growth on GDD. The data refer to the year 2008, which coincides with the average time of tissue formation of the retrospective growth observations as taken in 11^th^ NFI during 2009–2012.

**Table 1:** Land cover and forest sample size by regions.

Region	Land area		Forestry land	Forest land	Number of measured forest plots in 2009–2012
		1000 km^2^			
1	6.7		4.3	3.6	918
2	7.2		5.1	4.7	1066
3	16.9		10.9	9.8	2513
4	14.3		9.6	9.3	2380
5	10.5		7.7	7.4	1879
6	12.6		9.7	9.2	2300
7	14.3		12.6	12.2	3095
8	19		14.3	12.6	3034
9	16.7		14.4	13.8	3258
10	16.8		14	13.5	3197
11	17.8		15.8	14.6	3430
12	21.5		20.4	17	3264
13	35.5		31.3	24.8	4377
14	64.5		62.5	42.2	3998
15	28.1		27.9	7.8	567
Total	302.4		260.5	202.5	39276

‘Forestry land’ is a land use concept referring to lands with no other priority assigned except forestry. ‘Forest land’ is a land cover concept referring to lands, which are estimated to produce at least 1 m^3^ of wood per hectare and year as a long-term average. Data are from [12] and from METLA (A. Ihalainen, pers. comm.). While the location of the study area is the same as in [8] the geographical borders of regions slightly differ and, in particular, region 15 in the very north was not reported in [8].

The approach of this study is strictly empirical and is based on high-quality observations on daily temperature and forest growth, which in Finland are unique within the boreal biome. Measurements from the period 1961 to 2012 (to 2013 for GDD) were chosen, thus obtaining a sufficiently long period of time for trend analyses. Earlier records and reports were available referring to the period 1931–1960 and were used as a reference.

The earlier work [8], [9] addressed the same area in Finland and applied growth observations of Finland's 3^rd^ NFI, which was conducted in 1951–1953. Noting these sampling years and the time lag of retrospective monitoring, the analysis referred to the forest increment during the period 1946–1952. In the earlier papers [8], [9] GDD data of the climate normal period 1931–1960 were applied. The method was improved in this new analysis as follows. The kriging method, which was not yet available for [8], provides a better spatial representation of the temperature data. As the forested area varies between regions, the growth observations in regression analysis were weighed by the area of forest land (see Table 1). In determining the growth-GDD regression, the timing of the temperature and growth data was better matched (the observations referring to 2006–2011).

## Results

### Warming trends

**Figure 2:**
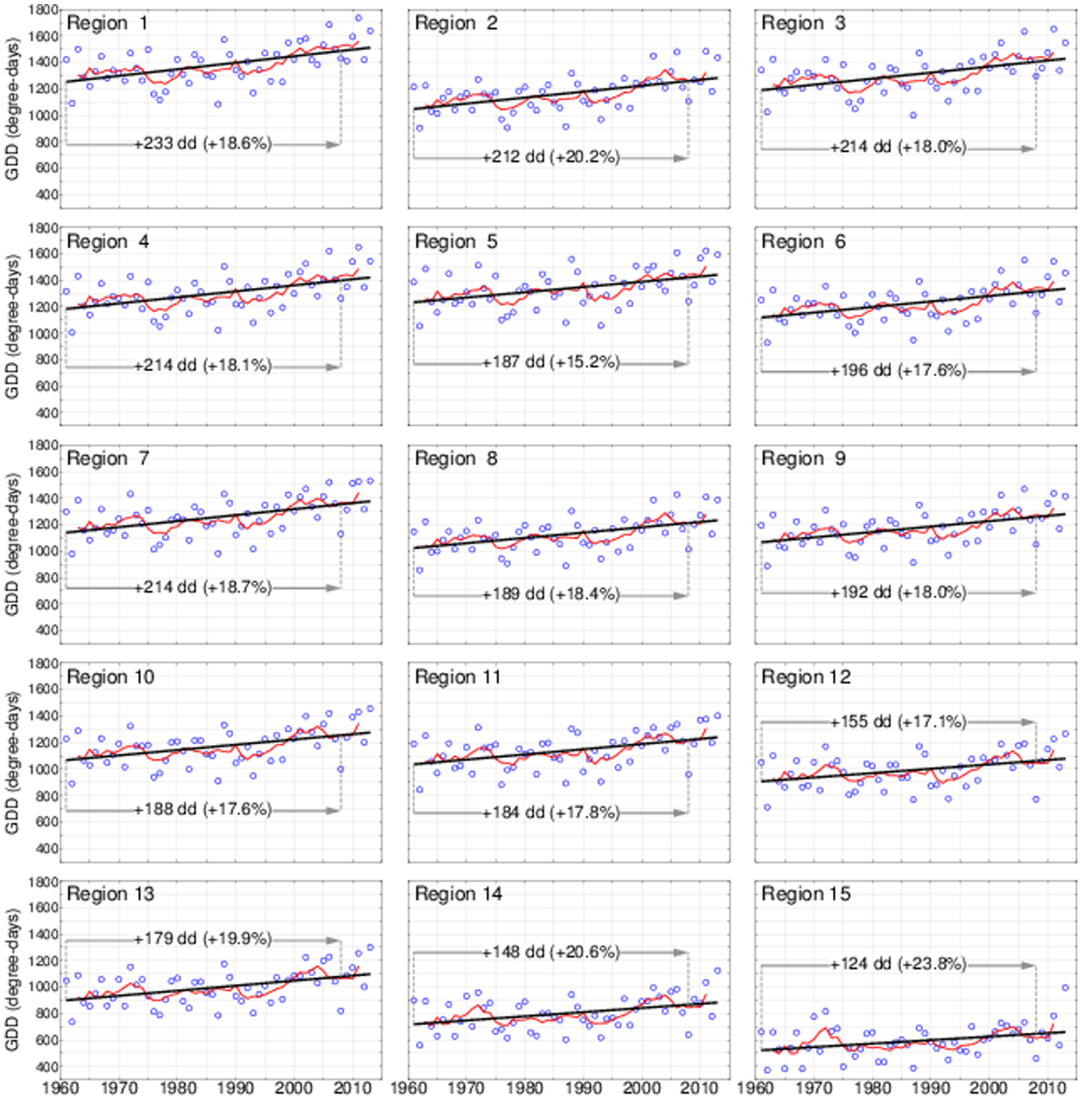
Annual Growing Degree Days (GDD) 1961–2013 for the regions (blue circles). Also shown are the linear regression lines (black) and the 5-year moving averages from 1963 to 2011 (red lines).

Climatic warming was quantified from the GDD regressions, comparing their estimates for the growing seasons of 1961 and 2008 (Fig. 2). The latter year coincided with the forest growth as measured in the 11^th^ NFI. Warming was clearly observed as the GDD was higher for 2008 than for 1961 in all regions. The GDD for 2008 (and 1961) in each region refer to the estimates as obtained from the time series regression, not GDD as measured in that particular year, nor the five-year moving average (see Fig. 2). The warming trends were statistically significant (p>95% in all regions). In absolute terms, the GDD warming was largest in the southernmost region 1 (+233 degree days) and smallest in the northernmost region 15 (+124 dd). However, in line with projections [9], the largest relative change was in the northernmost region 15 (+23.8%); while the smallest relative change occurred in the south-eastern region 5 (+15.2%). Comparing the moving averages with the regressions it appears that the warming in all regions was particularly significant since 1990. In all regions, the five-year average for the latest observed year 2011 is higher than the regression estimate for 2011 and for 2008 (Fig. 2).

### Impact of warming on growth

**Figure 3:**
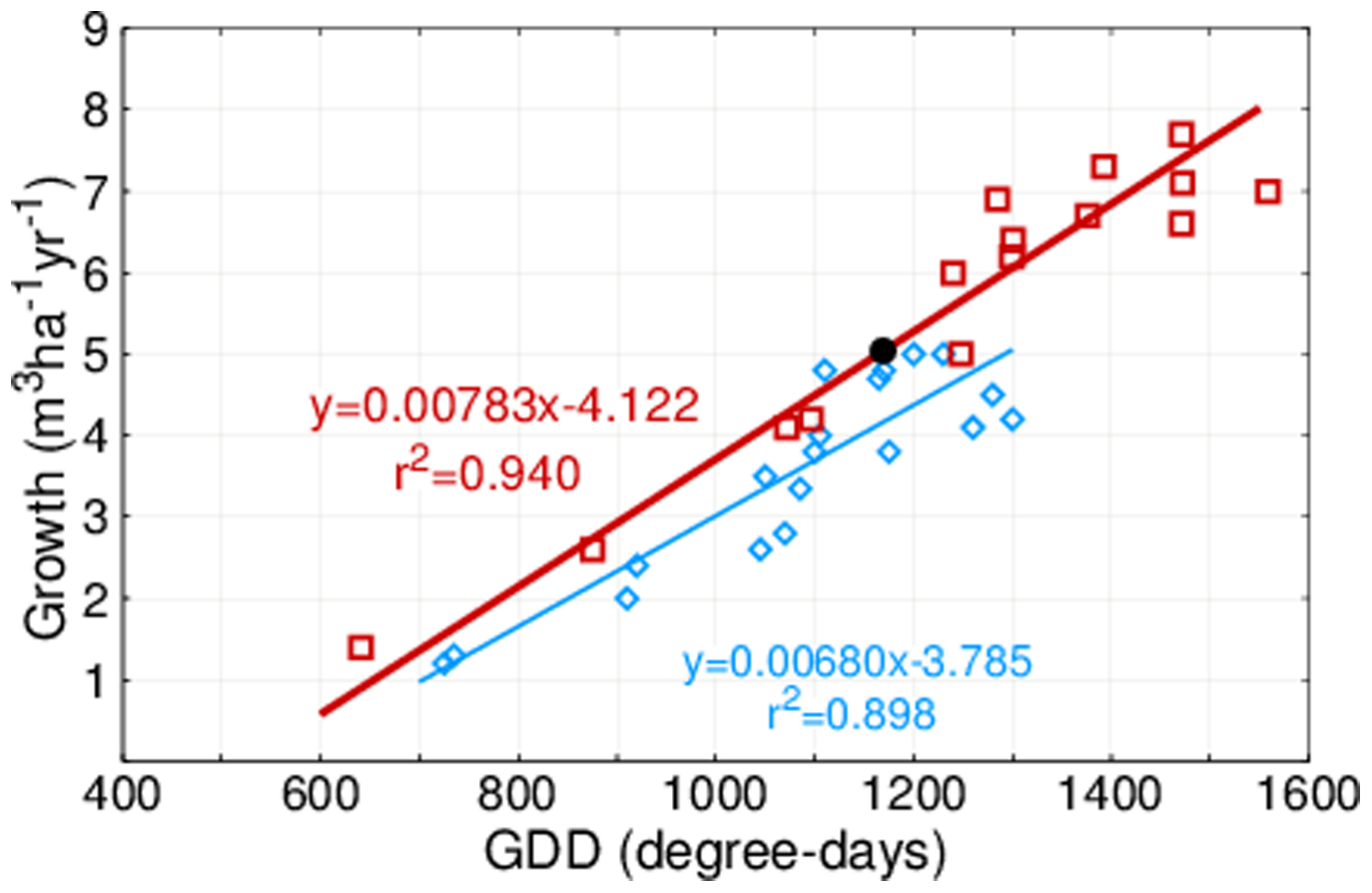
Regressions relating forest growth to Growing Degree Days. The new regression (red) referring to 2006–11 is based on data from the 15 regions shown in Figure 1. The black dot on the red line shows the area-weighted average of all 15 regions. The old regression (blue) as published in [8] was based on 19 data points as recorded in the mid-20^th^ century.

The observations of this study can be compared with the earlier results [8]. Relating the most recent growth observations of the 11^th^ NFI to the average GDD of 2006–11, a new regression was obtained with almost the same slope as earlier (Fig. 3). Differences between the results were as follows: (i) The new region 15 in the very north widened the range to lower values; (ii) The warming impact moved the data points to the right; and (iii) Other drivers affecting growth were reflected in the elevated position of the new regression above the old one.

Reproducing successfully the growth-to-GDD regression and obtaining an improved fit lends new support to using GDD in analyses of spatial and inter-annual variations of forest growth in the boreal zone. The new data were temporally better matched and spatially more representative than those reported earlier [8], and in the new analysis the growth estimates were weighted by the area of forest land. These improvements of the method and the inclusion of region 15 contributed to the better fit (r^2^ = 0.94).

GDD above 1,350 degree-days were common in many regions in 2006–11, unlike in the mid-20^th^ century (Figs. 2 and 3). However, warming was not the only driver affecting growth. Forest management, a gradual change in the species composition and age structure of forests, CO_2_ fertilization, improving water-use efficiency, nitrogen deposition and other (unknown) factors can have played a role. In the earlier projections a maximum growth of 6 m^3^ ha^–1 ^yr^–1^ was postulated, expecting drought limitations to come into play [9]. This turned out to be incorrect, as growth above 6 m^3^ ha^–1 ^yr^–1^ was now observed in the majority of regions, even though the area-weighted country average did not exceed 5 m^3^ ha^–1 ^yr^–1^. We cannot predict whether drought limitations will come into play in the future, especially as future climate may trigger changes in precipitation [17].

What would have been the growth of these boreal forests had the warming not occurred? This question was addressed in the following way: For every region we calculated the GDD for the years 1961 and 2008 from the regional regressions given in Fig. 2. Inserting the pairs of GDD values into the new GDD-growth regression (Fig. 3), we computed the warming impact on growth per hectare in every region as the difference of the two obtained growth values. This difference was multiplied by the forest area of each region (see Table 1), resulting in estimates of regional growth due to warming; and the difference to the overall growth was assigned to other factors (Table 2).

**Table 2:** Measured growth by regions, the warming impact, and the growth unrelated to warming.

Region	Mesured growth	Warming impact	Growth unrelated to warming
		*Million m^3^ yr^−1^*	
1	2.6	0.7	1.9
2	2.9	0.8	2,1
3	6.6	1.7	4.9
4	7.2	1.6	5.6
5	5.3	1.1	4.2
6	6.2	1.4	4.8
7	8.9	2.0	6.8
8	6.4	1.9	4.5
9	8.8	2.1	6.8
10	9.4	2.0	7.4
11	8.8	2.1	6.7
12	7.2	2.1	5.1
13	10.7	3.5	7.2
14	11.7	4.9	6.8
15	1.5	0.8	0.7
Total	103.9	28.4	75.6

All results refer to the year 2008, with estimates for 1961 used as reference.

A growth of 103.9 million m^3^ yr^−1^ was observed in the boreal forests of Finland referring to approximately 2008 as described in the 11^th^ NFI (Table 1, based on [12], excluding Ahvenanmaa (Åland)). This can be compared to the observed growth of 49.5 million m^3^ yr^−1^ as measure in the 4^th^ NFI in 1960–63 [18]. Warming-induced increment is estimated at 28.4 million m^3^ yr^−1^ (Table 2). An additional change of +26 million m^3^ yr^−1^ would hence be the response to other drivers, such as improved forest management, CO_2_ fertilization and other (unknown) mechanisms. Given uncertainties, we estimate that the growth of boreal forests of Finland would have been about 75 million m^3^ in 2008, had the growing season temperatures remained at the level of the 1960’s.

## Discussion and Conclusions

More than half of the significant growth improvement in the boreal forests of Finland has been a response to climatic warming. Such a large change in productivity has greatly affected the ecological and economic performance of these forests. The long interval of about 60 years and the large geographic scale from the northern to the southern edge of the boreal zone give confidence for obtaining conclusive results. The regression analyses were applied as they are simple and transparent and enable comparison with earlier results [8], [9].

The analysis in this paper addressed the statistical correlation between forest growth and growing degree days. There could be causal mechanisms affecting forest growth, which vary in time and space and are unrelated to temperature and, nevertheless, trigger similar spatial patterns as those depicted in Table 2. However, it has been well documented that the inter-annual variation of growth in the boreal zone correlates with changes in temperature. Moreover, the slope of the regression is reproduced using two independent data sets (see Fig. 3). Therefore, we conclude that the spatial and temporal variation of growing season temperature is the main causal factor affecting variations of forest growth in this region.

New research is needed to elaborate on the ecological and eco-physiological mechanisms by which increasing temperatures stimulate forest growth. An empirical analysis like this cannot characterize the mechanisms of such large impacts. It is possible that the various drivers reinforce one another based on synergistic mechanisms, thus creating an aggregated warming impact. Analogous attribution problems are met in plant sciences in assessing the relative effect of plant genotype and of the environment in affecting plant phenotype and yield [19]. Finland-specific land management, nitrogen deposition, or disturbance history may have affected the warming response. However, warming has been widely observed [10], CO_2_ concentration is spatially invariant and similar warming responses have been reported recently from Canada [20] and in Russia [21]. Indirect observations reinforce this view [4], [5]. Therefore, a significant warming impact on productivity has likely occurred more broadly in forest ecosystems of the boreal biome.

The growth concept that we applied is similar to “gross growth” as described in [11]. The fraction of gross growth that decomposes in the forest has been relatively small in Finland (national total <5 million m^3^ annually). Forest fires have been virtually non-existent; and trees, which have been damaged by storms or insects, are often harvested for industrial or energy use. The growth concept in the Finnish NFI has not changed since the 1960’s and, therefore, the time series are directly comparable.

Ecological and economic assets are at stake in Finnish forestry. For example, a major expansion plan of forest industries in Finland was recently announced, though not yet confirmed, drawing on the ample timber resources. The new plant would start operating in 2017 and would be located in region 6. If confirmed, the new plant is the largest single investment project in forest industry to date in Finland. The plant would process about 4 million m^3^ of round wood annually. Timber shortage would restrict such initiatives in absence of the warming impacts. More broadly, the implications of climatic warming are immensely diverse. The increment of woody biomass feeds into forest carbon sinks [22]. Research should address the climate feedbacks and assess how hydrology or forest albedo will change as forest growth and standing biomass gradually increase in the circumpolar boreal forests [23]–[26].

Papers in the 1980 s suggested that boreal forests are sensitive to climatic warming, should a warming occur [8], [9]. This study reports that a warming has occurred by 2013 and boreal forests have responded with accelerating growth. Although the data cover only Finland, similar responses have been reported based on research in other parts of northern hemisphere forests [21]. It is important to continue and expand the monitoring programs of both climatic changes and ecosystem responses, and to further improve their scientific quality.
